# Utilizing Internet Search Volume to Monitor Stages of Change in Vaccine Hesitancy During the COVID-19 Outbreaks

**DOI:** 10.3389/fpubh.2022.844543

**Published:** 2022-07-04

**Authors:** Yu-Tung Lan, Shiow-Ing Wu, Yu-Hsuan Lin

**Affiliations:** ^1^Department of Epidemiology, Harvard T.H. Chan School of Public Health, Boston, MA, United States; ^2^Institute of Population Health Sciences, National Health Research Institutes, Miaoli, Taiwan; ^3^Department of Psychiatry, National Taiwan University Hospital, Taipei, Taiwan; ^4^Department of Psychiatry, College of Medicine, National Taiwan University, Taipei, Taiwan; ^5^Institute of Health Behaviors and Community Sciences, College of Public Health, National Taiwan University, Taipei, Taiwan

**Keywords:** COVID-19, Google Trends, stages of change, vaccine hesitancy, vaccine willingness

## Abstract

Real-time vaccine hesitancy surveillance is needed to better understand changes in vaccination behaviors. We aim to understand the association between coronavirus disease 2019 (COVID-19) outbreaks and population vaccine hesitancy and to monitor the dynamic changes in vaccination behaviors. We used the autoregressive integrated moving average model to examine the association between daily internet search volume for vaccines and two waves of COVID-19 local outbreaks in Taiwan from 19 March to 25 May, 2021. During the small-scale outbreak, the search volume increased significantly for 7 out of 22 days with an average increase of 17.3% ± 10.7% from the expected search volume. During the large-scale outbreak, the search volume increased significantly for 14 out of 14 days, with an average increase of 58.4% ± 14.7%. There was a high correlation between the search volume and the number of domestic cases (*r* = 0.71, *P* < 0.001). Google Trends serves as a timely indicator to monitor the extent of population vaccine willingness.

## Introduction

Vaccination is critical to prevent serious illness and obtain herd immunity from coronavirus disease 2019 (COVID-19), yet vaccine hesitancy is a major threat to reaching those goals. In addition to identifying vaccine-hesitant people, there is a spectrum of hesitancy among those unvaccinated, ranging from resistant, through neutral and receptive to avidly seeking (1). This spectrum can be framed as a health-seeking behavior with different stages of change, including pre-contemplation, contemplation, and preparation—before finally taking action (2). Until late April 2021, Taiwan was often cited as a model of pandemic control despite its low vaccination rate; but beginning on 20 April, the country has been facing a mass transmission event. The dynamic changes in vaccine willingness demonstrated a longitudinal model different from previous findings reported in cross-sectional surveys.

Emerging studies based on infodemiology have shown to be a powerful tool in public health research (3–8). With the benefits of high temporal resolution and real-time collection of data, it is possible to monitor population health behaviors in a time-sensitive manner. A previous study had used internet search data to accurately estimate where and when the influenza outbreak would be (6). There was also a study using Google Trends to identify seasonal depression on a global scale (7). In addition, our previous multinational longitudinal study found that increased internet search for insomnia were an important indicator of mental health burden during the COVID-19 pandemic (8). In the current study, we aim to investigate the temporal association between the number of domestic COVID-19 cases and the vaccine willingness in Taiwan by internet search data.

## Methods

Daily confirmed COVID-19 cases, including domestic and imported cases, and the cumulative vaccination rate were obtained from the Taiwan Centers for Disease Control (9). Utilizing data from Google Trends, we used daily search for “疫苗” (*yimiao*), the Mandarin word for “vaccine,” from 19 March 2021 to 25 May 2021, as a surrogate for the Taiwanese vaccine willingness. This period includes three significant events: a vaccination campaign starting on 22 March, a small-scale local outbreak starting on 20 April, and a large-scale local outbreak beginning on 12 May. Then, we selected 3 months (19 December 2020 to 18 March 2021) before the vaccination campaign as the baseline for future search volume prediction. The 3-month baseline was selected to inform our prediction since a longer time window could be contaminated by other past relevant events. The search category was set to all categories.

Google Trends does not provide information on the absolute number of search. Instead, it provides a relative search value to display search activity for a given term according to a specific period, time, and area. Each data point is divided by the total search of the geography and time range it represents to compare relative popularity. This value is scaled from 0 to 100. A value of 100 is the peak popularity of the term, while a value of 50 means that the term is half as popular in a given period with search volumes for the days given relative to this.

We estimated the expected daily search volume using Hyndman and Khandakar's algorithm for the autoregressive integrated moving average (ARIMA) model (10). ARIMA model is a forecasting algorithm that uses time-series data to predict future trends. Using the daily search data from 19 December 2020 to 18 March 2021, we forecasted the expected daily search volume of a counterfactual scenario in which two waves of local outbreaks had not occurred from 19 March 2021 to 25 May 2021. We then examined the number of days with the observed daily search volume being higher than the upper limit of the 95% confidence interval of the expected search volume. In addition, Pearson's correlation coefficient test was performed to examine the association between daily search volume for “疫苗” and daily confirmed domestic and imported cases. Statistical analyses were performed using R software, version 3.6.1.

## Results

**Figure 1:**
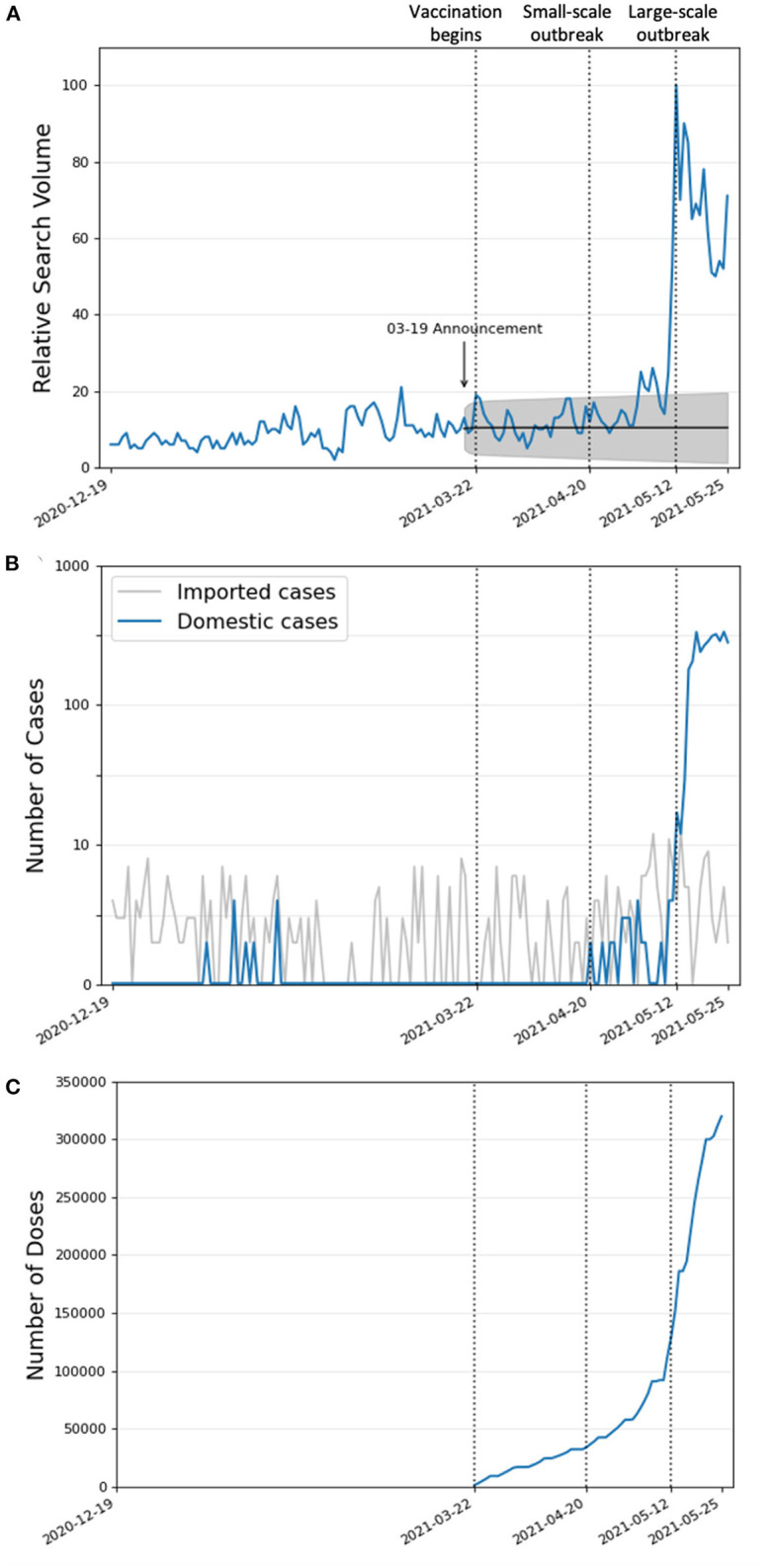
Internet search volume of “vaccine” in Taiwan from 19 December 2020 to 25 May 2021, the number of daily confirmed COVID-19 cases, and the cumulative vaccination doses administered during this time period. **(A)** The relative search volume for “vaccine”. The gray area indicates the 95% confidence interval of the expected search volume (black line). The first dotted line corresponds to the start of vaccination on March 22. The second dotted line corresponds to the start of the small-scale outbreak on April 20. The third dotted line corresponds to the start of the large-scale outbreak on May 12. **(B)** The log-transformed number of daily confirmed domestic (in blue) and imported (in gray) cases from 19 December 2020 to 25 May 2021. **(C)** The cumulative vaccination doses administered from 22 March 2021 (when vaccination began) to 25 May 2021.

The search volume and the number of domestic cases were highly correlated (*r* = 0.71, *P* < 0.001). Figure 1A shows that a low search volume for “疫苗” corresponds to the low number of domestic COVID-19 cases (Figure 1B) until April 20, 2021, even though vaccination began during this period. The search volume increased significantly for 7 out of 22 days during the small-scale outbreak (20 April to 11 May, 2021), with an average increase of 17.3 ± 10.7% from the expected search volume. During the large-scale outbreak (12–25 May), the search volume significantly increased for 14 out of 14 days, with an average increase of 58.4 ± 14.7% from the expected search volume. However, the search volume was less associated with the number of imported COVID-19 cases (*r* = 0.33, *P* = 0.006), which stayed mainly the same during the study period.

Figure 1C shows the cumulative vaccine doses administered in Taiwan by date. Before the two local outbreaks, fewer than 35,000 doses were administered. After the small-scale outbreak and before the large-scale outbreak, more than 95,000 doses were administered over 22 days. From the start of the large-scale outbreak until the end of the study (14 days), almost 190,000 doses were administered.

## Discussion

To the best of our knowledge, this is the first study using real-time population-level data to investigate the association between domestic outbreaks and population vaccine willingness. Our study found that Taiwanese vaccine willingness was associated with local outbreaks rather than the beginning of the vaccination campaign or imported COVID-19 cases. These findings suggest that the internet search volume for “疫苗” serves as a timely indicator to monitor the extent of population vaccine willingness and the dynamics through three time periods corresponding to Prochaska's behavior change model (2).

Being vaccinated is a health-seeking behavior and could be framed by the stage of change model to become vaccinated (11). During the first month of the vaccination campaign, the search volume and the vaccination rates were very low despite a sufficient amount of vaccines being available, indicating that most Taiwanese were at the stage of pre-contemplation. During the small-scale outbreak, the search volume and vaccination rates increased. However, less than one-third of the days in this period showed significantly higher search volume compared to the baseline. The top related query for vaccines on Google Trends was the side effects of vaccination. These search patterns suggest that the public was aware of the threat of COVID-19 and started to weigh the risks and benefits of vaccination but had not yet taken action during this contemplation stage. During the large-scale outbreak, there was a significant increase in the search volume for “疫苗”, as well as the national vaccination rates. The related queries, including vaccine appointments and information related to the AstraZeneca vaccine (the only vaccine available in Taiwan at that time), suggested that the public was in the preparation/action stage.

Vaccine hesitancy is a delay in acceptance or refusal of vaccination despite the availability of vaccination services (12). It can be influenced by factors such as complacency, confidence, and convenience. Thus, as risk perceptions increase, vaccine acceptance is likely to increase as well. Previous studies had found a positive association between the two (13, 14). In the current study, it is possible that Taiwanese' vaccine willingness increased due to other factors besides domestic confirmed cases; yet, given adequate vaccines in the first month of vaccination and the top related query alongside “疫苗”, it is more likely that vaccine willingness was related to local outbreaks.

This study utilizes daily internet search data to demonstrate the dynamic change in vaccine willingness through the two waves of COVID-19 local outbreaks. We show a higher temporal resolution compared to previous longitudinal studies, in which self-reported questionnaires with intervals of several weeks to months were the primary way to gain information (15). Therefore, our findings were able to sensitively detect changes in vaccine willingness through the two waves of outbreaks that happened within one month. In addition, the vaccine willingness was associated with the local outbreaks rather than the beginning of the national vaccination campaign or imported COVID-19 cases. This supports the idea that messaging to the public is more effective when addressing personal benefits (e.g., preventing serious illness) rather than collective benefits (e.g., obtaining herd immunity) (16). However, as Google Trends is a type of crowdsourcing data, it is inherent to the limitations raised by using these databases (17–20). First, the search for “疫苗” may not reflect the willingness to vaccination. Factors other than local outbreaks, such as the advertisement of vaccination on media and the reported side effects of vaccination, may influence internet search behaviors. Second, Google Trends does not represent a random sampling of the population and may exclude the vulnerable group with limited access to the internet. Third, since Google Trends represents population data, we were unable to determine the demographic and socioeconomic status of each conducting the search. Thus, crowdsourcing is not meant to replace but supplement traditional methods in public health surveillance. Nevertheless, the collective phenomenon of internet search behavior can be a meaningful tool to monitor vaccine willingness at the population level. Further research is needed to clarify the findings of this study, including the relationship between domestic cases and vaccination willingness and interventions to reduce vaccine hesitancy, based on the stages of change model.

## Data Availability Statement

The raw data supporting the conclusions of this article will be made available by the authors, without undue reservation.

## Author Contributions

Y-TL, Y-HL, and S-IW: conceptualized the study and analyzed and interpreted the data. Y-TL and Y-HL: drafted the manuscript. All authors have read and approved the final version of the manuscript.

## Funding

This study was supported by a grant from the National Health Research Institutes of Taiwan (11A1-PHGP13-052).

## Conflict of Interest

The authors declare that the research was conducted in the absence of any commercial or financial relationships that could be construed as a potential conflict of interest.

## Publisher's Note

All claims expressed in this article are solely those of the authors and do not necessarily represent those of their affiliated organizations, or those of the publisher, the editors and the reviewers. Any product that may be evaluated in this article, or claim that may be made by its manufacturer, is not guaranteed or endorsed by the publisher.
